# Competitive HRP-Linked Colorimetric Aptasensor for the Detection of Fumonisin B1 in Food based on Dual Biotin-Streptavidin Interaction

**DOI:** 10.3390/bios10040031

**Published:** 2020-03-30

**Authors:** Zui Tao, You Zhou, Xiang Li, Zhouping Wang

**Affiliations:** 1 State Key Laboratory of Food Science and Technology, Jiangnan University, Wuxi 214122, China; taozui@vip.jiangnan.edu.cn (Z.T.); zhouyouox@vip.jiangnan.edu.cn (Y.Z.); lixiang@njucm.edu.cn (X.L.); 2 School of Food Science and Technology, Jiangnan University, Wuxi 214122, China; 3 International Joint Laboratory on Food Safety, Jiangnan University, Wuxi 214122, China; 4 Collaborative Innovation Center of Food safety and Quality Control of Jiangsu Province, Jiangnan University, Wuxi 214122, China

**Keywords:** aptamer, fumonisin B1, mycotoxin, horseradish peroxidase, food safety

## Abstract

Fumonisin B1 (FB1) is the most prevalent and toxic form among fumonisin homologues which are produced by fusarium species and it contaminates various types of food products, posing serious health hazards for humans and animals. In this work, a colorimetric assay for the detection of FB1 has been developed based on competitive horseradish peroxidase (HRP)-linked aptamer and dual biotin-streptavidin interaction. In short, a biotinylated aptamer of FB1 was immobilized on the microplate by biotin-streptavidin binding; the complementary strand (csDNA) of the aptamer was ligated with HRP by biotin-streptavidin binding again to form a csDNA-HRP sensing probe, competing with FB1 to bind to the aptamer. The color change can be observed after the addition of chromogenic and stop solution, thereby realizing the visual detection of FB1. Under optimal conditions, good linearity was observed within the concentration range of 0.5 to 300 ng/mL, with a detection of limit of 0.3 ng/mL. This assay is further validated by spike recovery tests towards beer and corn samples, it provides a simple, sensitive and reliable method for the screening of FB1 in food samples and may be potentially used as an alternative to conventional assays.

## 1. Introduction

Fumonisin is produced in cereals by *Fusarium verticillioides*, *Fusarium proliferatum* and other related species due to fungal infection; it is among the most harmful mycotoxins which exert severe health hazards on humans and animals [1,2,3]. As of now, 28 fumonisin homologues have been discovered and characterized as fumonisin A, B, C and P, among which, fumonisin B1 (FB1) is the most prevalent and toxic form [4], it was classified as a group 2B possible carcinogen for humans by the International Agency for Research on Cancer (IARC) [5].

As a heat-stable, water-soluble mycotoxin, FB1 was isolated from moldy corn for the first time by Gelderblom et al. in 1988 [6]. The occurrence of FB1 has been reported in various countries all over the world [7,8,9,10], and it was detected in various crops including corn, sorghum, wheat and rice in all aspects of grain harvesting, storage, and processing as well as in agricultural products and cereal products such as raisins, dates, peanuts, nuts and flours [11,12,13,14]. In the beer production process, malting, milling and washing of barley and other grains may increase the level of FB1 [15,16,17,18]. Pascari et al. reviewed information on the incidence of mycotoxins in beer, and reported the occurrence of FB1 in beer of different productions such as craft beer, lager beer, sorghum beer and pale beer [17]. Notably, according to Campone et al., FB1 was the most widely distributed mycotoxin in analyzed beers (>21%) which were collected from Italian supermarkets [18].

Besides neurotoxicity and immunotoxicity, organ and tissue toxicity are the most hazardous characteristics of FB1 [19], which has severe toxic effects on liver, kidney and intestinal tissues, leading to liver and esophagus cancers in humans [20,21,22]. In the European Union, the maximum level of fumonisin (sum of FB1 and FB2) in maize-based foods for direct human consumption was set at 4 μg/kg by EC188l-2006.

Conventional detection methods of FB1 include thin layer chromatography (TLC) [23], liquid chromatograph-mass spectrometer-computer (LC-MS) [24], enzyme-linked immunosorbent assay (ELISA) [25] and high performance liquid chromatography (HPLC) [26]. These methods offer high sensitivity and specificity, while they often require costly and sophisticated instruments, specialized practitioners or stable sources of antibodies, which are time-consuming and labor-intensive.

In order to overcome these problems, various alternative techniques have been developed. For instance, Lu et al. developed an electrochemical immunosensing method by disposable screen-printed carbon electrode for the detection of FB1, with the linear range of 0.2–4.5 ppm [27]. Shu et al. proposed a chemiluminescence ELISA method for detection of FB1 which employed an Ab2b-Nb-AP fusion protein as bio-reporter, the detection of limit was 0.35 ng/mL [28]. Peltomaa et al. used epitope mimics to develop a microarray-based immunoassay, with a dynamic range from 17.3–79.6 ng/mL [29].

Among these methods, aptamer based sensing systems showed promising performance in detection. Aptamer is a single-stranded oligonucleotide selected from a random oligonucleotide library by SELEX technology [30]. Under certain physical and chemical conditions, aptamer can be folded into hairpins, neck rings and other structures which specifically bind to target analytes [31]. Compared with traditional antigen and antibody technologies, aptamers have the advantages of a wide range of target substances, easy access, stable properties and easy preservation with higher selectivity, specificity and affinity, and are easily modified with various functional groups, and thus connected to a solid phase carrier, which can be used as a capture probe for analysis and detection [32,33,34]. In recent years, a large number of detection methods based on aptamer recognition of heavy metal ions [35], foodborne pathogens [36] and mycotoxins [37] have been reported. Sheng et al. used light-up RNA aptamers to develop a transcription amplified aptasensor for detecting Staphylococcus aureus with the dynamic range of 10^2^–10^6^ CFU/mL [38]. Lv et al. developed an aptamer-based sensing platform of single-walled carbon nanohorns for Ochratoxin A detection with a linear detection range of 20–500 nmol/L [39].

Taking into account the above-mentioned facts, in this work, a colorimetric assay for the detection of FB1 based on competitive HRP-linked aptamers and dual biotin-streptavidin interactions was established for the first time. In short, biotin-labeled FB1 aptamers were immobilized on the microplate, the complementary strand DNA (csDNA)-HRP sensing probes and the analyte were added into the microplate simultaneously, competing to bind to the FB1 aptamer. Then, chromogenic solution and stop solution were added; the absorbance of the solution at 450 nm was measured with a microplate reader. This assay is simple, sensitive and reliable for FB1 sensing application in food.

## 2. Materials and Methods

### 2.1. Chemicals and Apparatus

Standards of Fumonisin B1 (FB1), Aflatoxin B1 (AFB1), Zearalenone (ZEN), Deoxyniv1enol (DON) and Ochratoxin A (OTA) as well as streptavidin were purchased from Sigma-Aldrich LLC (Shanghai, China). Na_2_HPO_4_, KH_2_PO_4_, KCl, NaCl, Na_2_CO_3_, NaHCO_3_, Tween-20, CH_3_OH, H_2_O_2_, Bovine Serum Albumin (BSA) were obtained from Sino-pharm Chemical Reagent Co., Ltd (Shanghai, China), Fumonisin B1 ELISA detection kit was purchased from Pribolab Co., Ltd (Beijing, China); biotin, Streptavidin-HRP and TMB Chromogenic Reagent kits were obtained from Sangon Biotech Co. Ltd. (Shanghai, China). Ultrapure water used throughout this work was purified by a Milli-Q water purification system. Absorbance values were collected by a Synergy H1 Hybrid Multi-Mode Microplate Reader (BioTek Instruments Inc., Winooski, VT, USA). The oligonucleotides sequences were synthesized by Sangon Biotech Co. Ltd. (Shanghai, China) according to Derosa et al.’s report [40] and their sequences were as follows:

Biotin-labeled FB1 aptamer sequence (ssDNA): 5′-biotin-ATA CCA GCT TAT TCA ATT AAT CGC ATT ACC TTA TAC CAG CTT ATT CAA TTA CGT CTG CAC ATA CCA GCT TAT TCA ATT AGA TAG TAA GTG CAA TCT-3′;

Biotin-labeled complementary strand sequence (csDNA): 5′-biotin- AGA TTG CAC TTA CTA TCT AAT TGA ATA AGC TGG TAT GTG CAG ACG TAA TTG AAT AAG CTG GTA TAA GGT AAT GCG ATT AAT TGA ATA AGC TGG TAT -3′.

### 2.2. Preparation of the Complementary Strand-HRP Sensing Probe

Firstly, streptavidin-modified HRP was diluted, specifically, 2 μL of HRP (1 mg/mL), and 998 mL of 1 × PBS buffer (10 mmol/L Na_2_HPO_4_, 2 mmol/L KH_2_PO_4_, 2.7 mmol/L KCl, 137 mmol/L NaCl, pH 7.4) was added to obtain HRP diluent; then 996 μL of HRP diluent and 8 μL of biotin-modified complementary strand (10 μmol/L ) were mixed evenly, incubated at 10 °C for 3 h, before the csDNA-HRP sensing probes were obtained and stored at −20 °C.

### 2.3. Preparation of a Streptavidin-Coated Microplate

Firstly, streptavidin was diluted with carbonate buffer (15 mmol/L Na_2_CO_3_, 35 mmol/L NaHCO_3_, pH 9.6). Streptavidin diluent (200 μL) was added to each well and incubated in a refrigerator at 4 °C for 12 h, then the solution was poured out quickly, and the microplate was washed 3 times with PBS-T washing buffer (containing 0.05% Tween 20 in 1 × PBS buffer). To prevent non-specific adsorption, which may lead to adverse effects towards the results, 200 μL of BSA blocking solution was added to the microplate. Microwells that were not completely coated with streptavidin were blocked, placed in a shaker at 37 °C for 1 h (150 r/min), then the plate was washed 3 times with PBS-T washing buffer, and finally a streptavidin-coated microplate was obtained.

### 2.4. Detection Procedure

Biotin-modified FB1 aptamer (10 μL) was added into a streptavidin-coated microplate and incubated for 30 min at 37 °C in a shaker (150 r/min). The solution was poured out quickly, and the microplate was washed 3 times with PBS-T washing buffer (gently shaking for 1 min each time). The washing solution was then removed, and the plate patted dry. To the microplate, 100 μL of the analyte and 100 μL of the csDNA-HRP sensing probe was added, evenly mixed, and incubated for 1 h at 25 °C. The solution was poured out quickly, and the microplate was washed 3 times with PBS-T washing buffer (gently shaking for 1 min each time) and patted dry. Finally, 100 μL of TMB chromogenic solution was added to each microwell. After being kept at room temperature for 10 min, 100 μL of 2% H_2_SO_4_ stop solution was added, and the absorbance of the solution at 450 nm was measured with a microplate reader.

### 2.5. Preparation and Measurement of Food Samples

To verify the accuracy and feasibility of the proposed aptasensor in practical applications, beer and corn samples purchased from a local supermarket were used for spiked recovery experiments. These samples were prepared as follows: the beer sample was placed in a refrigerator at 4 °C for 30 min, and then the gas in the beer was completely removed by ultrasonication. 20 g of the beer sample was weighed and dissolved in a 50 mL volumetric flask containing 70% methanol, and 10 mL of the solution was weighed, diluted 5 times with ultrapure water, and then filtered through a 0.22 μm filter membrane. On the other hand, 20 g of crushed corn sample was dissolved in a beaker containing 50 mL of 70% methanol, stirred at high speed for 2 min and filtered with quantitative filter paper; 10 mL of the filtrate was weighed, diluted 5 times with ultrapure water, and then filtered through a 0.22 μm filter membrane. Three different concentrations of FB1 standards (10 ng/mL, 50 ng/mL, 100 ng/mL) were added to the beer and corn samples, respectively. Spiked samples (100 μL) of different concentrations were measured by this method and ELISA kits separately.

## 3. Results and Discussion

### 3.1. Principle of the Detection of FB1 by the Aptasensor

**Figure 1:**
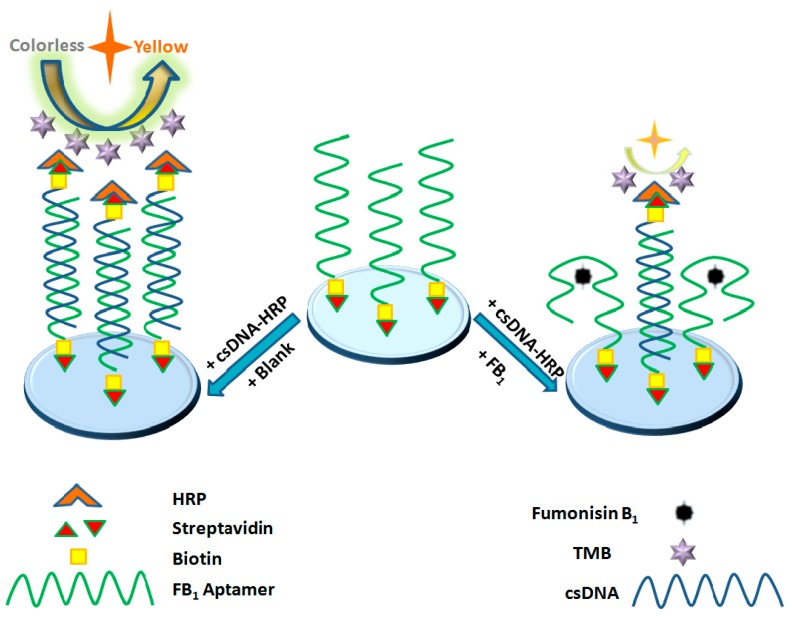
Principle of the detection of Fumonisin B1 (FB1) by the aptasensor.

FB1 aptamers can specifically recognize and bind FB1, and can be used as capture probes to detect target molecules in analytes. The biotin-labeled FB1 aptamer was immobilized on a streptavidin-coated microplate, and the complementary strand of the aptamer was connected to HRP to form a complementary strand-HRP composite, serving as a sensing probe. The csDNA-HRP composite competed with the target analyte to bind to the aptamer. TMB chromogenic solution was added (under the catalysis of HRP), the colorless TMB solution was oxidized to oxTMB, the color then changed to blue, before sulfuric acid stop solution was added, and the solution turned yellow. The concentration of target analyte was inversely proportional to the color shade. When the analyte contained FB1, only a small number of csDNA-HRP sensing probes were connected to the aptamer, and the color of the solution was light yellow; when the analyte did not contain FB1, a large number of csDNA-HRP sensing probes were connected to the aptamer, and the color of solution was dark yellow. By measuring the absorbance of the solution at 450 nm and observing the change in the color of the solution, the visual detection of FB1 in the analyte can be achieved. The principle of detection is shown in Figure 1.

### 3.2. Optimization of Test Conditions

**Figure 2:**
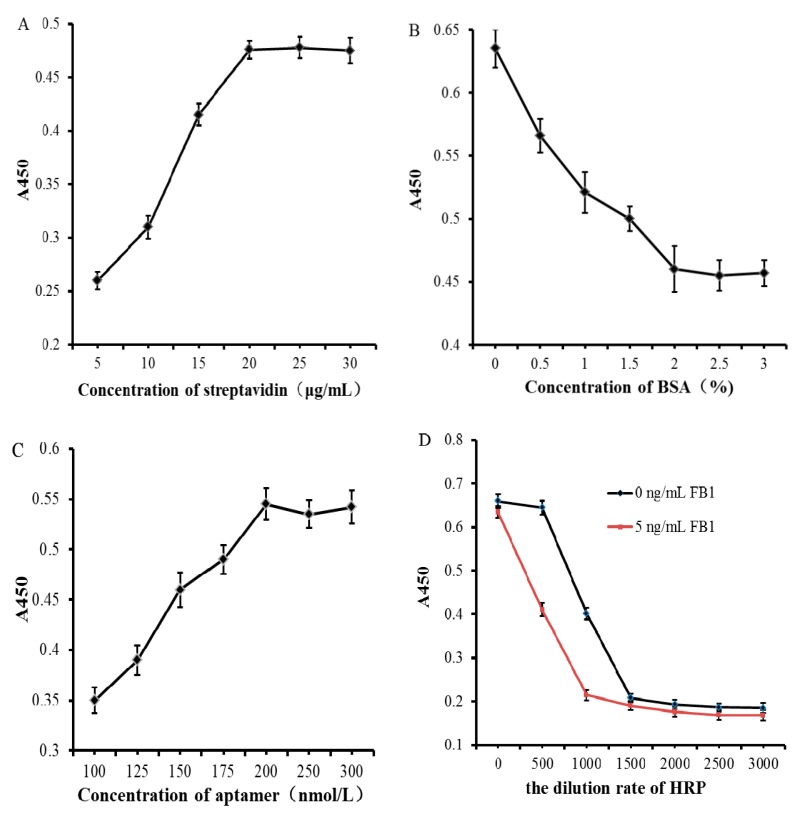
(**A**) Optimization for the concentration of streptavidin. (**B**) Optimization for the concentration of BSA. (**C**) Optimization for the concentration of FB1 aptamer. (**D**) Optimization for the dilution rate of streptavidin–horseradish peroxidase (HRP).

To achieve the ideal performance of the proposed method, four parameters including concentration of the streptavidin, concentration of the BSA blocking solution, concentration of the FB1 aptamer and the dilution rate of streptavidin-HRP were optimized. Biotin-labeled FB1 aptamers were immobilized on the microplate by biotin-streptavidin binding, the number of aptamers was determined by the amount of streptavidin coated on the bottom of the microplate, which had a great impact on the detection sensitivity. In this work, six different concentrations of streptavidin were selected; the absorbance value of the blank sample at 450 nm was measured. As shown in Figure 2A, in the concentration range of 5 μg/mL to 20 μg/mL, as the concentration increased, the color of the solution in the microplates gradually deepened; as the streptavidin concentration reached 20 μg/mL, the A450 value no longer increased, and the color of the solution in the microplate no longer darkened, indicating that the amount of streptavidin coated with the microplate reached a saturated state. Therefore, 20 μg/mL was selected as the optimal coating concentration of streptavidin.

In this work, streptavidin-labeled HRP may be adsorbed on the bottom of the micro wells which are not coated with streptavidin; the results of detection would be affected by this kind of non-specific adsorption. To avoid such a situation, BSA blocking solution was added to block the streptavidin-coated microplate, while with a low concentration of BSA blocking solution, the non-specific adsorption still occurs. Six different concentrations of BSA blocking solutions were selected for optimization (0.5%, 1%, 1.5%, 2%, 2.5%, and 3%). The absorbance value of the blank sample at 450 nm was measured. As shown in Figure 2B, within the concentration range of 0% to 2%, as the concentration of the BSA blocking solution increased, the color of the solution in the microwell gradually lightened, and the absorbance value of A450 also continued to decrease, indicating that the non-specific adsorption was continuously reduced. When the concentration of BSA blocking solution reached 2%, the color of the solution in the microplate no longer lightened and the absorbance value of A450 did not decrease. This indicates that the BSA blocking solution has completely blocked the part of the bottom of the microplate that was not coated with streptavidin. Therefore, 2% BSA was employed.

As a biosensor, biotin-modified FB1 aptamer can specifically recognize and capture target FB1, in this work, the aptamer hybridized with the csDNA-HRP signal probe to achieve visual detection of FB1. The concentration of FB1 aptamer has a greater impact on the results. Seven different concentrations of FB1 aptamers were selected for optimization. The absorbance value of the blank sample at 450 nm was measured. As shown in Figure 2C, as the concentration of aptamers increased from 100 nmol/L to 200 nmol/L, the amount of csDNA immobilized to the bottom of the microplate and the amount of HRP introduced into the detection system also increased, the A450 value increased steadily and the color of the solution became dark. When the aptamer concentration reached 200 nmol/L, the color of the solution in the microplate no longer darkened, and the absorbance value did not increase, indicating that the concentration of the aptamer reached the saturation state. Thus, 200 nmol/L was selected as the optimal concentration.

The csDNA-HRP composite was used as a signal probe in this work. HRP can catalyze the oxidation of TMB to produce the reaction product which is blue. H_2_SO_4_ stop solution was added, the color turned yellow. The color of the solution was inversely proportional to the concentration of FB1 in the analyte. The higher the concentration, the lighter the color. To improve the sensitivity of the aptasensor, FB1 containing samples (5 ng/mL) and blank samples were detected simultaneously. When the difference between the absorbance values of the two kinds of samples reached the maximum, that is, the difference of color discrimination reached the maximum, the corresponding HRP dilution rate was optimal. Seven different dilution rates were selected for optimization (1:100, 1:500, 1:1000, 1:1500, 1:2000, 1:2500, and 1:3000). Target analytes and blank samples were detected simultaneously. As shown in Figure 2D, the absorbance of the blank sample was higher than that of the sample containing FB1. As the HRP dilution rate increased, the signal intensity of the solution gradually decreased. When the HRP dilution rate was 500 times, the difference between the absorbance of the FB1-containing sample and the blank sample at 450 nm reached the maximum, realizing the best colorimetric effect. Therefore, the optimal HRP dilution rate was 500 times.

### 3.3. Detection Performances

**Figure 3:**
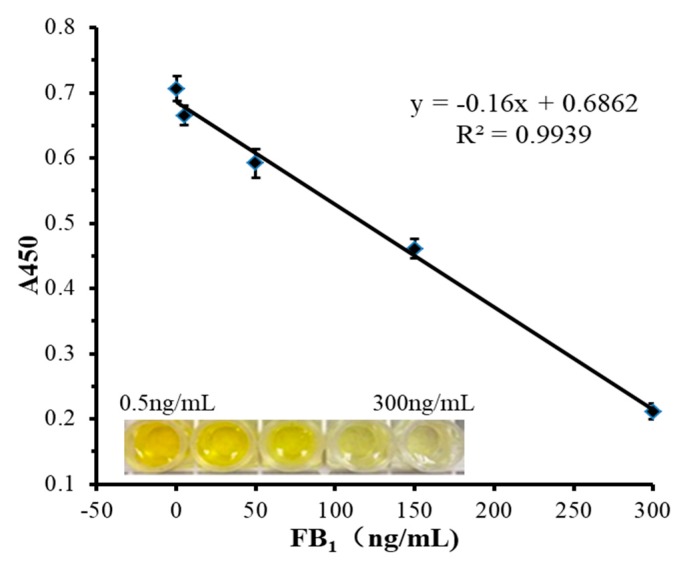
The linear relationship between FB1 concentration and the absorbance value at 450 nm.

Under the optimal conditions, competitive HRP-linked aptamers were used to detect different concentrations of FB1. When FB1 did not exist in the system, the color intensity of the csDNA-HRP sensing probe reached the maximum, and the color of the solution was dark yellow. As the concentration of FB1 increased, FB1 and csDNA-HRP sensing probes compete with the aptamer. The binding rate between the csDNA-HRP sensing probe and the aptamer gradually decreased, the intensity of the color signal correspondingly weakened, the color of the solution gradually lightened, and the A450 absorbance value also gradually decreased. As shown in Figure 3, the concentration of FB1 had a clear linear relationship in the range of 0.5–300 ng/mL (R^2^ = 0.9939), with the limit of detection of 0.3 ng/mL which was calculated based on the 3σ rule. The proposed method was compared with other FB1 detection methods reported in recent years, as shown in Table 1. The linear range and the limit of detection of this method was considered to be competitive among those other listed methods. Besides, this method had no time-consuming steps, the dual biotin-streptavidin interaction was easy to achieve, and the color change could be distinguished by naked eyes, which provides a simple and flexible approach to FB1 detection.

### 3.4. Analysis for the Specificity

**Figure 4:**
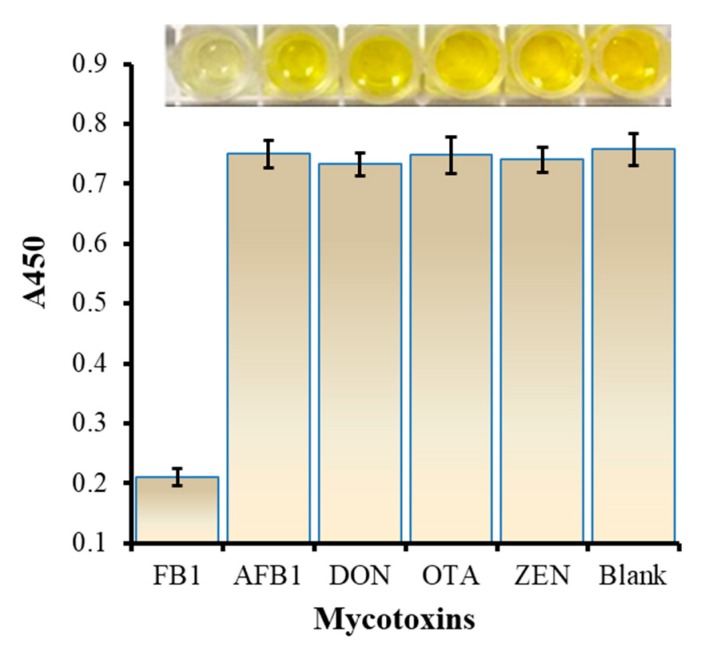
The evaluation for specificity of the proposed method.

To verify the specificity of the proposed method, four mycotoxins including ZEN, DON, AFB1 and OTA were introduced and detected with FB1 and a blank sample by this method under optimized conditions at a concentration of 300 ng/mL. As indicated in Figure 4, compared with the other mycotoxins and blank samples, the A450 absorbance value of the FB1 sample was lower and the color was lighter. This indicates that this proposed method has selectivity towards FB1.

### 3.5. Analysis for the Stability

**Figure 5:**
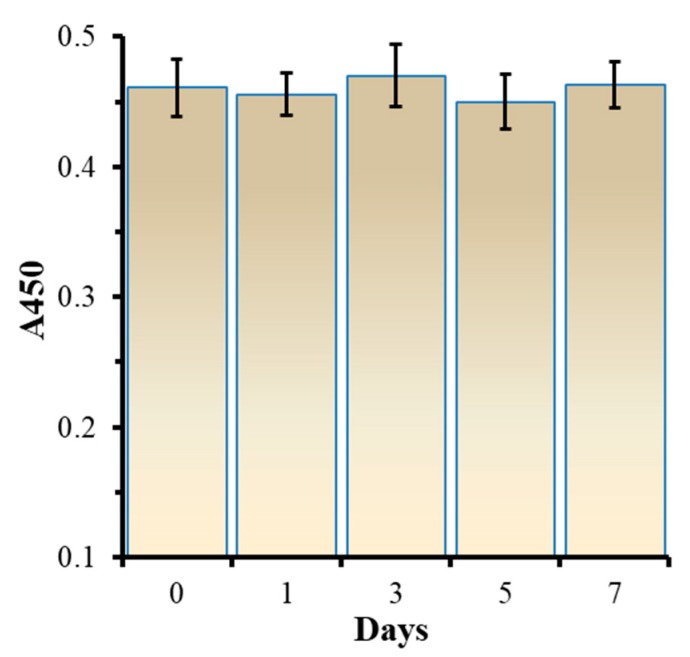
The evaluation for stability of the proposed method.

To verify the stability of the developed method, the FB1 aptamers were immobilized into microplates and stored in a refrigerator at 4 °C; repeated detections of FB1 were carried out after 1 day, 3 days, 5 days and 7 days by the method under optimized conditions at a concentration of 150 ng/mL. As shown in Figure 5, A450 absorbance values were basically the same throughout 7 days of analysis, indicating that the method has good repeatability and stability.

### 3.6. Determination of FB1 in Spiked Food Samples

To verify the feasibility and accuracy of this method, the beer and corn samples purchased from a local supermarket were pre-processed, then three different concentrations of FB1 standard solutions were added to the two kinds of samples, and a spike recovery test was performed by the aptasensor and ELISA kits. This method which uses aptamers as the recognition probe can overcome some limitations of antibodies in preparation, immunization and modification, as well as avoiding the shortages of strict store and reaction conditions of the kits. As shown in Table 2 and Table 3, the recovery rate of beer samples was 90.10% to 102.2%, and the recovery rate of corn samples was 91.80% to 105.2%. The experimental results are consistent with the ELISA method, which certifies this proposed method has great accuracy and stability, showing the potential for use in the detection of FB1 in different food samples.

## 4. Conclusions

In this work, a colorimetric detection method for fumonisin B1 based on a competitive HRP-linked aptamer was developed. An aptamer capable of specifically recognizing and capturing fumonisin B1 was immobilized on the microplate by biotin-streptavidin binding as a molecular recognition element, and the csDNA of the aptamer was connected to HRP by biotin-streptavidin binding again to catalyze the color reaction of TMB. Under the optimal conditions, the absorbance of the solution at 450 nm has a clear linear relationship with the concentration of FB1. The detection linear range is 0.5–300 ng/mL, with a limit of detection of 0.3 ng/mL. It is proved that this aptamer assay shows high specificity towards FB1. Moreover, the developed assay is capable of complex sample sensing, this method and ELISA kits were used to perform spike recovery experiments towards beer and corn samples. The results show that the proposed method is simple, sensitive and reliable in practical applications, and can be used as an alternative for conventional assays towards the detection of fumonisin B1 in food samples in rapid screening and other fields.

## Figures and Tables

**Table 1:** Comparison of different detection methods towards FB1.

Detection Method	LOD (ng/mL)	Linear Range (ng/mL)	Reference
SPE based Electrochemical Immunosensor	4.2	200–4500	[27]
Chemiluminescence ELISA	0.35	0.93–7.73	[28]
Microarray-Based Immunoassay	11.1	17.3–79.6	[29]
AuNPs based dc-pELISA	3.07	3.125–25	[41]
Nanotubes based Electrochemical sensor	0.002	0.01–1000	[42]
Colloidal gold immunoassay	2.5	2.5–10	[43]
Aptamer based colorimetric assay	0.3	0.5–300	this work

**Table 2:** Detection and recovery results of FB1 in beer samples by the developed method and enzyme-linked immunosorbent assay (ELISA) kits (n = 3).

Number		Background (ng/mL)	Added (ng/mL)	Found (ELISA) (ng/mL)	Spike Recovery (ELISA) (%)	Found (ng/mL)	Spike Recovery (%)
beer	1	0	10	9.140	91.40	9.010	90.10
	2	0	50	50.68	101.4	51.08	102.2
	3	0	200	197.4	98.69	197.2	98.60

**Table 3:** Detection and recovery results of FB1 in corn samples by the developed method and ELISA kits (n = 3).

Number		Background (µg/kg)	Added (µg/kg)	Found (ELISA) (µg/kg)	Spike Recovery (ELISA) (%)	Found (µg/kg)	Spike Recovery (%)
**corn**	1	0	10	9.260	92.60	9.180	91.80
	2	0	50	52.21	104.4	52.60	105.2
	3	0	200	206.3	103.1	207.3	103.7
